# Cloning and expression of an active aspartic proteinase from *Mucor circinelloides* in *Pichia pastoris*

**DOI:** 10.1186/1471-2180-13-250

**Published:** 2013-11-09

**Authors:** Jose Antonio Gama Salgado, Martin Kangwa, Marcelo Fernandez-Lahore

**Affiliations:** 1 School of Engineering and Science, Jacobs University Bremen, Campus Ring 1, Bremen 28759, Germany

**Keywords:** Aspartic proteinase, *Mucor circinelloides*, *Pichia pastoris*, SMART™ RACE PCR (Rapid Amplification of cDNA Ends), Alpha factor secretion signal, Milk clotting units (MCU)

## Abstract

**Background:**

Extracellular aspartic proteinase (MCAP) produced by *Mucor circinelloides* in solid state fermentations has been shown to possess milk clotting activity and represents a potential replacement for bovine chymosin in cheese manufacturing. Despite its prospects in the dairy industry, the molecular characteristics of this enzyme remain unknown. This work focuses on MCAP cloning and optimization of heterologous expression in *Pichia pastoris*, and characterization of the enzyme.

**Results:**

The cloning of cDNA sequence encoding MCAP from *M. circinelloides* was performed using a fragment of approximately 1 kbp as a probe. The fragment was amplified using non-specific primers designed from the NDIEYYG and KNNYVVFN consensus motifs from aspartic proteinases of different fungi. Gene specific primers were designed to amplify a full-length cDNA using SMART™ RACE PCR*.* MCAP was expressed in *P. pastoris* under the control of the constitutive GAP promoter. It was shown that *P. pastoris* secreted non-glycosylated and glycosylated MCAPs with molecular weights of 33 and 37 kDa, respectively.

**Conclusion:**

A novel MCAP was expressed in *P. pastoris* and efficiently secreted into the culture medium. The expression of the heterologous proteins was significantly increased due to advantages in codon usage as compared to other expression systems. The results suggest that *P. pastoris* could be exploited as a safe production platform for the milk clotting enzyme.

## Background

Rennet, a milk coagulant from the abomasum of milk-fed calves and lambs, is the industrial gold standard in cheese manufacturing [1]. Chymosin is the major milk-clotting component of natural rennet preparations obtained from young animals as its activity amounts to 90% of the total observed potency [2]. However, due to increased demand in cheese products, animal-derived milk coagulants are not sufficient to cover the production. Therefore, the demand has prompted increased research efforts in the manufacture of recombinant and microbial rennin [3]. Nevertheless, the rennin of microbial origin might be contaminated by other enzymes which might affect cheese ripening by causing bitterness during storage [4].

Until now, several aspartic proteinases (APs) such as pepsin, rennin, renin, and cathepsin D have been extensively studied. Microbial APs such as rhizopuspepsin and penicillopepsin have been reported to be either intracellular or extracellular enzymes with most of them having been cloned and purified. For example, acid proteinase from *Metschnikowia reukaufii*[5] has been cloned and expressed in *Escherichia coli* while three *clt* genes encoding milk-clotting proteinases from *Myxococcus xanthus* have been cloned and expressed in *E. coli*, *Saccharomyces cerevisiae* and *P. pastoris*[3].

It is also known that fungal extracellular thermophilic proteinases from *R. miehei* and *R. pusillus* are still used as substitutes for calf chymosin in cheese manufacturing [6]. However, the enzymes are extensively proteolytic which may result in impaired cheese organoleptic characteristics. Moreover, the proteinases are resistant to heat treatment compared to bovine chymosin and thus can remain active in the curd for longer periods of time [7]. Additionally, proteinases with low heat stability have been observed in *M. varians*[8] and *M. circinelloides*[9]*.* Although studies on the characterization of an acid proteinase from *M. circinelloides* were performed, the molecular characteristics of the enzyme remain unknown. The mentioned thermo-labile proteinases may provide technological advantages for industrial utilization. In this work, the cloning and expression of the aspartic proteinase from *M. circinelloides* was performed.

## Methods

### Fungal strain, bacterial strains and plasmids

The microorganism used as the source of the gene encoding MCAP was *M. circinelloides* strain DSM 2183 (Deutsche Sammlung von Mikroorganismen und Zellkulturen GmbH, Braunschweig, Germany). *E. coli* strain TOP10 [10] was used to amplify the plasmids carrying the cloned gene. *E. coli* strains were grown overnight in Luria-Bertani medium (10 g L^-1^, tryptone, 5 g L^-1^ yeast extract, 5 g L^-1^ NaCl) at 37°C, 220 rpm. *P. pastoris* was grown in YPD medium (10 g L^-1^ yeast extract, 20 g L^-1^ peptone, 20 g L^- 1^ glucose) at 30°C for 3 days with shaking at 250 rpm. When required, the final antibiotics concentration for ampicillin was 100 μg mL^-1^ while for zeocin it was either 30, 50 or 100 μg mL^-1^. Plasmid pGAPZα-A (Invitrogen, Darmstadt, Germany) was used as the cloning and expression vector. Table 1 shows the plasmids and strains used in this study.

**Table 1:** List of microorganisms and plasmids used in this study

**Strain or plasmid**	**Genotype**	**Reference**
**Strains**		
***Escherichia coli***		
TOP10	F- mcrA Δ(mrr-hsdRMS-mcrBC) φ80lacZΔM15 ΔlacX74 recA1 araD139 Δ(ara-leu) 7697 galU galK rpsL (StrR) endA1 nupG	10
***Pichia pastoris***		
X-33	Wild type	Invitrogen
***Mucor circinelloides***		
DSM 2183	Wild type	German resource centre for biological material
**Plasmids**		
pGAPZα-A	The pGAPZα-A vector use the GAP promoter to constitutively express recombinant proteins in *Pichia pastoris*. Contains the zeocin resistance gene (*Sh ble*).	Invitrogen
pGAPZα+MCAP	pGAPZα-A derivative carrying the whole *MCAP* gene^1^.	This work
pGAPZα+MCAP-2	pGAPZα-A derivative carrying the *MCAP* gene without an intron^1^.	This work
pGAPZα+MCAP-3	pGAPZα-A derivative carrying the *MCAP* gene without an intron^2^.	This work
pGAPZα+MCAP-5	pGAPZα-A derivative carrying the *MCAP* gene without a signal sequence and without an intron^2^.	This work
pGAPZα+MCAP SP-1	pGAPZα-A derivative carrying from the amino acid sequence number 67 to 394 of the *MCAP* gene without an intron^1^.	This work
pGAPZα+SyMCAP-6	pGAPZα-A derivative carrying the *MCAP* gene without signal sequence and without intron. The original *MCAP* gene was adapted to the optimal codon usage of P. pastoris. The insert was cloned flush with the Kex2 cleavage site and in frame of the α- factor signal sequence and in frame with the C-terminal polyhistidine tag into the *Xho*I and *Not*I site of the pGAPZα-A.	This work

^1^The insert was cloned in frame with the α-factor signal sequence and in frame with the C-terminal polyhistidine tag into the *EcoR*I and *Not*I sites of the pGAPZα-A.

^2^The insert was cloned flush with the *Kex2* cleavage site and in a frame of the α-factor signal sequence and in a frame with the C-terminal polyhistidine tag into the *Xho*I and *Not*I site of the pGAPZα-A.

### Genomic DNA extraction

For genomic DNA extraction, *M. circinelloides* DSM 2183 spores (1 × 10^5^ spores) were inoculated into potato dextrose agar plates (PDA) which were incubated at 24°C for 3 days. The PDA medium was prepared according to the supplier’s protocol (Difco, Detroit, MI, USA). About 250 mg of fresh mycelium were collected with tweezers in a 1.5 mL vial. The mycelium were washed with sterile water and centrifuged at 5000 *g* for 2 min. The spores were lysed in 466 μL TE buffer (10 mM Tris-Cl, pH 8.0, 1 mM EDTA) with 3 μL Proteinase K (20 mg mL^-1^), 1 μL RNAse (10 mg mL^-1^) and 30 μL SDS (100 mg mL^-1^). The suspension was mixed gently to avoid shearing the chromosomal DNA, followed by incubation at 37°C for 1 h. The precipitated DNA was collected by centrifugation (15000 *g*, 10 min at 4°C), followed by phenol-chloroform extraction and ethanol precipitation as described [11].

### DNA manipulation

Restriction enzymes (*EcoR*I, *Xho*I, *Not*I and *Avr*II), T4 DNA ligase and *Taq* DNA polymerase were purchased from New England Biolabs (Frankfurt, Germany). All enzymes were used under the conditions specified by the manufacturer. Plasmids were isolated using a QIAprep Spin Miniprep Kit (QIAGEN, Hilden, Germany), and the PCR products were purified with the QIAquick PCR Purification Kit (QIAGEN, Hilden, Germany). PCR reactions were performed in a (total volume of 50 μL) Mastercycler ep gradient S (Eppendorf, Hamburg, Germany). The recovered PCR fragments and plasmids were sequenced by Eurofins MWG Operon (Ebersberg, Germany). Plasmids were transformed into *E. coli* and *P. pastoris* using a Multiporator (Eppendorf, Hamburg, Germany), according to the supplier’s protocol.

### Total RNA isolation

To obtain the full-length cDNA of *MCAP* gene, total RNA was isolated from solid-state culture of the *M. circinelloides* as follows: 250 mL Erlenmeyer flasks containing 10 g of wheat bran moistened with 200 mM HCl, up to a water content of 120% on a dry basis, and autoclaved at 121°C for 20 min, were inoculated with 5×10^6^ spores of *M. circinelloides*. Cultured for four days at 24°C, 100 mg of the mycelium were collected with tweezers and immediately used for total RNA extraction using the RNeasy Plant Mini Kit (QIAGEN, Hilden, Germany). The concentration and quality of the total RNA was determined by using the NanoDrop ND-1000 spectrophotometer (NanoDrop Technologies, Inc. Wilmington, Delaware, USA).

### First-strand cDNA synthesis, 5′-RACE cDNA and 3′-RACE cDNA

Two microgram of total RNA were used for the synthesis of the first strand of 5′-RACE-Ready cDNA and 3′-RACE-Ready cDNA. The synthesized first strand cDNA was used as a template for the 5′-RACE cDNA and 3′-RACE cDNA using the gene specific reverse primer GSP-Mucor-2R and forward primer GSP-Mucor-1 F, respectively (Table 2). In these cases, the conditions for PCR reactions were as described by Clontech (SMART RACE cDNA Amplification Kit User Manual). The amplified RACE fragments were separated by agarose gel electrophoresis and recovered using NucleoTrap Gel Extraction Trial Kit (Takara Europe-Clontech, Saint-Germain-en-Laye, France). Using this technique, the sequences of the extreme ends of the *MCAP* gene (5′and 3′) were obtained. Finally, the full-length cDNA sequence of the aspartic proteinase of *M. circinelloides* (deposited in GenBank under accession number JQ906105) was amplified from the 5′-RACE-Ready cDNA while the genomic *MCAP* of the aspartic proteinase (deposited in GenBank under accession number JQ906106) was amplified from genomic DNA of *M. circinelloides* using the forward primers APMC-Met-F and the reverse primer APMC-stop-R (Table 2).

**Table 2:** Oligonucleotides used for PCR amplification in this study

**Primer name**	**Sequence (5′-3′)**
Primers for DNA fragment PCR to obtain a partial sequence of genomic DNA of the acid proteinase	
12 ND-F	AACGATATCGAGTACTATGGT
M.cir-2R	TTAAAGACTTCATAGTTGTTCTT
Primer for 3′-RACE PCR (Gene-specific primer)	
GSP-Mucor-1 F	GATGGTCGTGCCTGGTCTATCCAAT
Primer for 5′-RACE PCR (gene-specific primer)	
GSP-Mucor-2R	CATTGTCTCTGGCACCGTATTGAGCAGC
Primers for full-length cDNA and recombinant plasmids	
APMC-EcoNaeI-F	ATGGAATTCGCCGGCGCTACTACTGATGCCACTGGTACTGTCCCCG
APMC-F	AGGAATTCTTCTCATTAGTCTCTTCTTG
APMC-Met-F	ATGGAATTCATGAAATTCTCATTAGTCTCTTCTTGTGTC
MCAP-3 F	TATCTCGAG*aaaaga*GCTCCCAGTGGTAGCAAGAA
XhoI-N-MCAP-F	TATCTCGAGaaaagaATGAAATTCTCATTAGTCTCTTCTTGTG
APMC-NotI-R	AAAGCGGCCGCGACAGATTTGGCAATTT
APMC-Stop-R	GTGATTTATAGATAGATAGATGAAATGTACCAAA
Primers to identify clones containing recombinant plasmids	
pGAP-F	GTCCCTATTTCAATCAATTGAACAAC
AOX1pGAP-Rev	CAAATGGCATTCTGACATCCTC

The underlined sequences (GAATTC; *Eco*RI, CTCGAG; *Xho*I and GCGGCCGC; *Not*I) represent the additional restriction sites at the 5′ ends of forward and reverse primers. The lowercase letters indicate the *Kex2* cleavage sites. The primers (for First-strand cDNA synthesis, 3′-RACE cDNA and 5′-RACE cDNA**)** provided in the SMART RACE cDNA Amplification Kit (Clontech) are not described in the table.

The PCR reactions contained the following components each listed at their final concentrations: 1 × Advantage 2 PCR Buffer, 200 pmol μL^-1^ dNTPs, 2 pmol μL^-1^ of each primer (forward and reverse), 2.5 μL of 5′ first-strand cDNA (unknown concentration), 1 × Advantage 2 Polymerase Mix (Clontech, Palo Alto, CA, USA). PCR was carried out at an annealing temperature of 61°C.

### Amplification of the cDNA encoding MCAP

**Figure 1:**
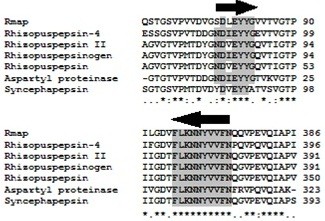
**Multiple alignment of the consensus motifs sequences NDIEYYG and FLKNNYVVFN of several fungal aspartic proteinases.** Consensus motifs sequences are marked in black arrows. Asterisks indicate conserved amino acids. The number to the right of the amino acid sequence is based on the protein.

To clone the full-length cDNA encoding MCAP in *M. circinelloides*, a partial sequence of genomic DNA of the acidic proteinase gene was first obtained. Non-specific primers (12 ND-F and M.cir-2R) (Table 2) were designed using the conserved motifs of aspartic proteinases from different species of filamentous fungi (Figure 1). In this case, the amino acid sequence of the *Mucor bacilliformis* proteinase [12] and those of *Rhizopus microspores* var*. rhizopodiformis* (accession number CAA72511), *Rhizopus niveus* (accession number Q03700), *Rhizopus microspores* var*. chinensis* (accession number AAB59306), *Rhizopus microsporus* var*. chinensis* (accession number AAA33881), *Rhizopus microsporus* var*. chinensis* (accession number AAA33879) and *Syncephalastrum racemosum* (accession number AAC69517) were downloaded from the GenBank and aligned with BLAST.

After PCR, a 956 bp fragment was obtained. PCR amplification was carried out at an annealing temperature of 52°C using 1.25 U *Taq* DNA polymerase and 200 ng of genomic DNA. The deduced amino acid sequence of the product was aligned with the amino acid sequence of aspartic proteinase from *M. bacilliformis*[12]*, R. niveus* (accession number X56992) and *R. microsporus* var. *chinensis* (accession number M63451) using BLAST algorithm. Since the fragment sequence showed high similarity to the selected proteinases, gene-specific primers were designed to perform 5′-RACE and 3′-RACE as well as for the amplification of a full-length cDNA of the aspartic proteinase gene from the first strand 5′-RACE-Ready cDNA of *M. circinelloides* by SMART™ RACE PCR Kit (Takara Europe-Clontech, Saint-Germain-en-Laye, France).

### Recombinant plasmids construction and codon usage adaptation

A set of expression plasmids were constructed by cloning a partial *MCAP,* whole *MCAP,* or *SyMCAP* gene in frame with the alpha-factor (α-MF) secretion signal and the C-terminal polyhistidine tag (6x His tag) into the multiple cloning site of pGAPZα-A, indicating that all *MCAP* products were cloned downstream of the glyceraldehyde-3-phosphate dehydrogenase (GAP) promoter [13]. The whole *MCAP* coding sequence (with intron sequence) was amplified from *M. circinelloides* genomic DNA while the full-length cDNA (without intron) or partial sequence cDNA (without signal peptide and without intron) encoding MCAP was amplified from the 5′ of the first strand cDNA. The final concentrations of components for PCR of recombinant plasmids was: 1 × ThermoPol reaction buffer, 200 pmol μL^-1^ dNTPs, 2 pmol μL^-1^ of each primer, 1 ng μL^-1^ plasmid DNA, 0.04 units μL^-1^*Taq* DNA polymerase. The first round of PCR amplification was carried out at 63°C for 5 cycles, and the second round of amplification was at 66°C for 25 cycles. To construct the plasmids pGAPZα+MCAP, pGAPZα+MCAP-2, pGAPZα+MCAP-SP1, pGAPZα+MCAP-3 and pGAPZα+MCAP-5, the PCR reactions were carried out using the following forward primers: APMC-F, APMC-Met-F, APMC-EcoNaeI-F, XhoI-N-MCAP-F and MCAP-3 F, respectively. While the reverse primer APMC-NotI-R was used in all the PCR reactions (Table 2). The PCR products were purified as previously described and were digested using restriction enzymes for which specific sites had been previously added using primers. The digested PCR products were then ligated into the appropriate sites of the multiple cloning site of pGAPZα-A using T4 DNA ligase. Additionally, original *MCAP* was adapted to the optimal codon usage of *P. pastoris* and cloned in frame with DNA sequence for the N-terminal α- factor signal sequence, under the GAP promoter (performed by MWG Operon, Ebersberg, Germany). The final plasmid construct was designated as pGAPZα+SyMCAP-6. The ligated products were transformed into electrocompetent *E. coli* cells with further selection in LB-zeocin plates and expression was performed using *P. pastoris* X-33.

### Transformation of recombinant plasmids containing *MCAP* gene into *P. pastoris*

To examine the expression of *MCAP* constructs in *P. pastoris* under the control of GAP promoter, recombinant plasmids were digested by *Avr*II restriction enzyme and transformed into *P. pastoris* competent cells (Invitrogen, Darmstadt, Germany). Eighty microlitres of *P. pastoris* cells were mixed with 2.5 μg of linearized recombinant plasmids. The transformation mixture (100 μL) was plated on YPD agar plates supplemented with zeocin (100 μg mL^-1^) and incubated at 30°C for 4 days. In order to confirm that *P. pastoris* contained the recombinant plasmid, PCR and sequence analysis were performed as previously described.

### Production of crude extracellular MCAP

For the production of MCAP in *P. pastoris*, starter cultures of single colonies of transformants were grown in 25 mL YPD media in 100 mL shake flasks for 20 h at 30°C. The cultures were inoculated in triplicate in 75 mL YPD in 250 mL shake flasks to a starting OD_600_ of 0.1. Cultivation was carried out for 4 days. Considering that glucose concentrations above 40 g L^-1^ did not show any increase in MCAP activity, enzyme expression was performed in 20 and 40 g L^-1^ glucose and adjusted to an initial pH of 5.0 and 7 with citric acid. In order to analyze the effect of temperature in the culture medium on MCAP expression, recombinants were grown at 23, 24, 25, 27 and 30°C, at initial pH of 5.0. The supernatant from cultures was taken every 24 h and cells were harvested by centrifugation at 4000 *g* at 4°C. Thereafter, milk clotting enzyme activity was analyzed in the supernatant broths. The supernatant culture from wild type *P. pastoris* was used as a negative control.

To analyse MCAP production by *M. circinelloides,* 6 day cultivation was performed in solid-state reactor. The crude extract was obtained according to the method of Areces and coworker [7] and assayed daily in duplicate. The obtained protein was considered as a control reference MCAP.

### Protein determination

The amount of protein in the crude extract, supernatant broth, as well in the chromatographic fractions was determined according to the Bradford procedure [14] and bovine serum albumin served as a standard (Fischer Scientific, Schwerte, Germany).

### Chromatographic analysis of MCAP

All chromatographic experiments were done using an ÄKTA purifier system (GE Healthcare, Munich, Germany). After removal of the cells by centrifugation at 4000 *g*, 4°C, he MCAP recombinant protein was purified from the supernatant by cation-exchange chromatography using a 5 mL HiTrap SP FF column attached to the ÄKTA purifier. The protein extract was adjusted to pH 3.1 using citric acid, and then a range of 37–48 mL of the mixture was injected to the previously equilibrated column with 50 mM citric acid buffer pH 3.5 and 75 mM NaCl. After washing with the same buffer and 75 mM NaCl, the elution was performed with the same buffer and 200 mM NaCl and step gradient was developed in 5 column volumes with a flow rate of 1 mL min^-1^. For protein content and milk clotting assays, 2.5 mL of chromatographic fractions were collected and analyzed.

### SDS-PAGE analysis

The crude extracellular supernatant proteins were desalted using a PD-10 column (GE Healthcare, Munich, Germany) equilibrated with 20 mM phosphate buffer at pH 7.2. These recombinant products were about 10 times concentrated at room temperature using Vacuum Concentrator 5305 (Eppendorf, Hamburg, Germany) and applied to a 12.5% SDS-PAGE. Purified enzyme and crude control reference MCAP were loaded directly into the SDS-PAGE gel and stained with Coomassie Brilliant Blue.

### Milk clotting assay

The milk clotting activity was analyzed according to the method of Arima and coworkers, with some modifications [15]. Initially, 1 mL of substrate made of 100 g L^-1^ skimmed milk powder and 10 mM CaCl_2_ in distilled water was added to a 10 mL test tube and the contents were incubated at 35°C for 10 min. Afterwards, 0.1 mL of enzyme sample was added to the pre-incubated substrate. One milk clotting unit (MCU) was defined as the enzyme amount which clotted 1 mL of the substrate within 40 min at 35°C [15]. Based on this definition, the clotting activity was calculated according to equation of Rao and coworker [16], (Equation 1).

(1) $$\mathrm{MCU}\;{\mathrm{mL}}^{‒1}=2\;400/\left(t.E\right)$$

where 2400 is the conversion of 40 min to s, t; clotting time (s) and E; the enzyme volume (mL).

### Deglycosylation assay

About 35 μg of crude extracellular protein from the recombinant X-33/pGAPZα+MCAP-5 cultivated in YPD medium at initial pH of 5.0 was digested with 2 units of endoglycosidase H (endo H) (New England Biolabs, Frankfurt, Germany) at 37°C for 2 h. The crude protein had previously been desalted using a PD-10 column and equilibrated with 20 mM phosphate buffer, pH 6.0.

### Proteolytic activity assay

Proteolytic activities (PA) of obtained chromatographic fractions were measured by the method of Fan and coworkers using N,N-dimethylcasein (DCM) as the substrate [17]. For the assay, 10 mg of DCM was dissolved in 1 mL of 20 mM phosphate buffer, pH 5.8. Subsequently, 45 μL of the solution was thoroughly mixed with 45 μL of enzyme sample and incubated at 35°C for 30 minutes. The reaction was stopped using 1.35 mL of 10% ice-cold trichloroacetic acid (TCA). The reaction sample was kept on ice for 30 min and later centrifuged at 15000 *g* for 15 min. The absorbance of the mixture was measured at 280 nm. To make the reference solution, TCA was added before the enzyme. One unit of proteolytic activity (U mL^-1^) was defined as the amount in microgram of tyrosine released from DCM per minute at 35°C. The extinction for tyrosine was taken as 0.005 mL μg^-1^ cm, (Equation 2).

(2) $$\begin{array}{l} \mathrm{PA}\;(\left(U/\mathrm{ml}\right)=\left(A_{280\;\mathrm{nm}}/0.005\right)\times 1.44\;V\\ \;\;\;\;\times \left(1/30\right)\times 1000/45) \end{array}$$

where V is volume in mL.

## Results and discussion

### Isolation of the partial *MCAP* gene

The gene encoding MCAP was amplified by PCR from *M. circinelloides* strain DSM 2183. A 959 bp fragment was amplified using primers designed based on homology against NDIEYYG and KNNYVVFN consensus motifs from aspartic proteinase of various species of filamentous fungi (Figure 1). The deduced amino acid sequence of the obtained 959 bp fragment indicated the presence of catalytic Asp residues found in most known aspartic proteinases. In aspartic proteinases, the catalytic Asp residues is found within the motif Asp–Xaa–Gly in which Xaa can be either Ser or Thr [18].

### Cloning and gene comparison of the cDNA encoding the acidic proteinase

After obtaining the partial DNA sequence of *MCAP*, specific primers were designed for the amplification of 3′-RACE and 5′-RACE of aspartic proteinase gene from the first-strand cDNA of *M. circinelloides* by SMART™ RACE PCR. The full-length cDNA of the aspartic proteinase from *M. circinelloides* was amplified from the 5′ first-strand, while the full-length *MCAP* encoding the aspartic proteinase was amplified from genomic DNA of *M. circinelloides*.

**Figure 2:**
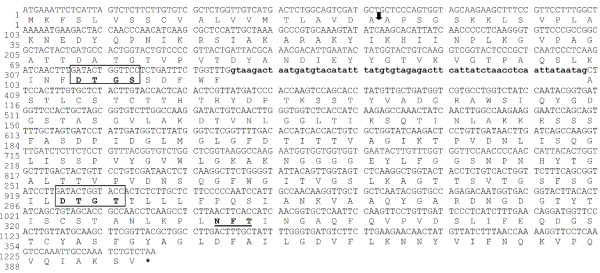
**The Nucleotide and deduced amino acid sequence of MCAP protein.** The deduced amino acid sequence is shown under the nucleotide sequence. The arrow indicates the proposed signal peptide cleavage site and lowercase letters indicate nucleotides in the intron sequence. The proposed catalytic Asp residues (motifs DTGS and DTGT) are boxed. The potential N-glycosylation site is underlined. Asterisk indicates the position of the stop codon (TAA).

By comparing the nucleotide sequence of aspartic proteinase amplified from the 5′first-strand cDNA with the sequence amplified from the genomic DNA of *M. circinelloides*, we found that the whole MCAP has an intron of 63 bp long and encodes 394 amino acid residues (Figure 2). The amino acid sequence of *M. circinelloides *MCAP was further aligned with the *M. bacilliformis*[12] sequence and with non-redundant protein database using BLASTX 2.2.24. The highest similarity between the MCAP amino acid sequence and a *M. bacilliformis* homolog was found to be 88% identity. The identity with *R. oryzae* (accession number ACL68088), *R. microsporus* (accession number CAA72511), *R. microsporus* var. *chinensis* (accession number AAB59305), *R. niveus (*accession number CAA40284), and *S. racemosum* (accession number AAC69517) was 66, 65, 64, 63, and 59%, respectively.

### Signal peptide sequence and N-glycosylation site

The analysis of the amino acid sequence by a SignalP 3.0 server identified a cleavage signal sequence site between positions Ala21 and Ala22 in the MCAP protein (http://www.cbs.dtu.dk/services/SignalP/). The putative signal peptide corresponding to the first 21 amino acids; MKFSLVSSCVALVVMTLAVDA, shows features of signal peptides, such as a highly hydrophobic region. Additionally, by using the NetNGlyc v1.0 server (Center for Biological Sequence Analysis, Technical University of Denmark DTU), one potential N-glycosylation site; Asn–X–Ser/Thr, was found to be at positions Asn331 in the MCAP (Figure 2).

### Protein expression, purification and SDS-PAGE analysis

**Figure 3:**
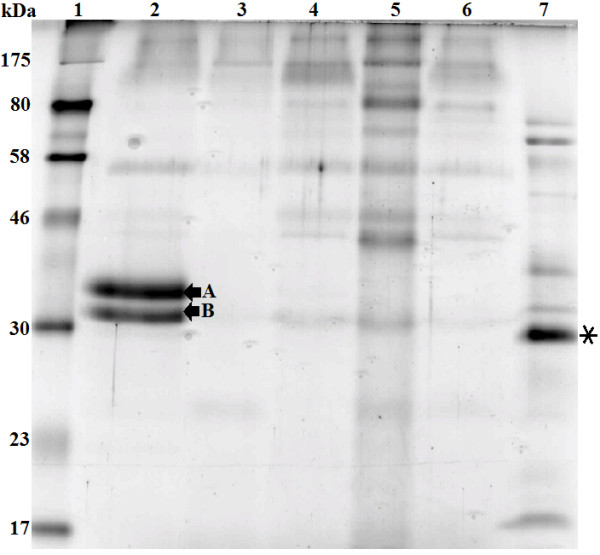
**SDS-PAGE analysis of the extracellular extract from recombinants X-33/pGAPZα +MCAP-2, X-33/pGAPZα+MCAP-3, X-33/pGAPZα+MCAP-5, X-33/pGAPZα+MCAP-SP1, *M. circinelloides *and *P. pastoris *X-33 (wild-type).** 25 μg of the concentrated protein products were subjected on each lane of SDS-PAGE. Samples: Lane 1, molecular standards (kDa); lane 2, secreted expression from recombinant X-33/pGAPZα+MCAP-5; lane 3, *P. pastoris* X-33 (negative control); lane 4, X-33/pGAPZα+MCAP-2; lane 5, X-33/pGAPZα+MCAP-3; lane 6, X-33/pGAPZα+MCAP-SP1; and lane 7, secreted expression from *M. circinelloides*. The asterisk indicates the authentic MCAP. The arrows indicate the expressed forms **(A and B)** of MCAP protein.

**Figure 4:**
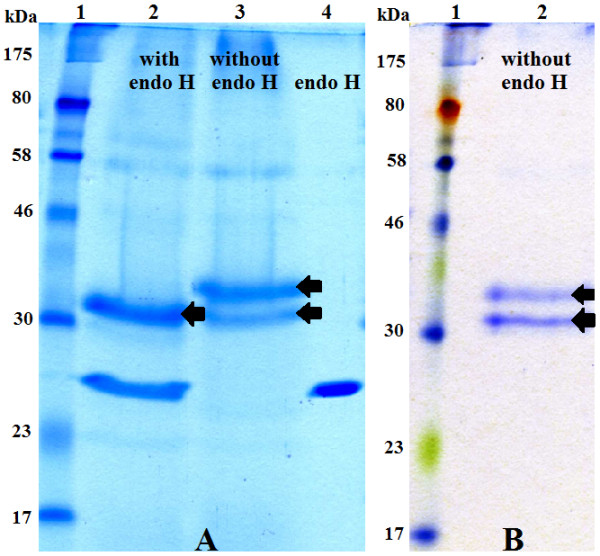
**SDS-PAGE electrophoretic pattern comparisons of recombinant *P. pastoris*. (A)** Enzymatic analysis of the MCAP protein with endoglycosidase (Endo H). 25 μg of the protein products were digested with endo H and subjected to SDS-PAGE. Lane 1, molecular standards; lane 2, secreted expression from X-33/pGAPZα+MCAP-5 (digested); lane 3, secreted expression from X-33/pGAPZα+MCAP-5 (undigested); lane 4, endo H. The arrows indicate the expressed forms of MCAP protein (above N-glycosylated protein, below the deglycosylated protein, respectively). **(B)** Analysis of the purified MCAP protein on HiTrap SP Sepharose Fast Flow. Lane 1, molecular standards; lane 2, 10 μg of secreted expression from recombinant X-33/pGAPZα+SyMCAP-6. The arrows indicate the expressed forms of MCAP protein (above N-glycosylated protein, below the deglycosylated protein, respectively).

**Figure 5:**
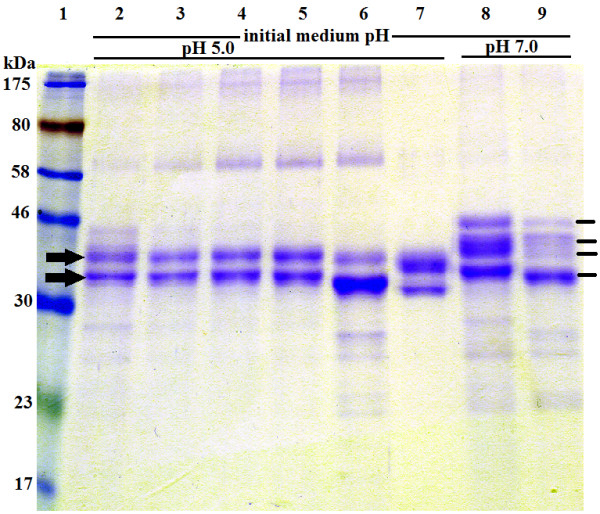
**Kinetics and forms of MCAP secreted by recombinant X-33/pGAPZα+MCAP-5 and X-33/pGAPZα+SyMCAP-6.** Recombinants were cultured for 24, 48, 72 and 96 hours in YPD medium (initial medium pH: 5.0 and 7.0) at 24°C. Proteins in the sample corresponding to 37 μL of the original supernatant broth were loaded on each lane of SDS-PAGE. Samples: Lane 1, molecular standards (kDa); lanes 2, 3, 4, 5, and 8, secreted expression from recombinant X-33/pGAPZα+MCAP-5 (lane 2, 24 h; lane 3, 48 h; lane 4, 72 h; lane 5, 96 h; lane 8, 72 h); lanes 6, 7, and 9, secreted expression from recombinant X-33/pGAPZα+SyMCAP-6 after 72 hours of cultivation. Lane 7, 4 μg of purified MCAP protein on HiTrap SP Sepharose Fast Flow. The arrows indicate the expressed forms of MCAP protein when the initial pH value of the medium was 5.0 and the lines indicate the expressed forms of MCAP at initial pH of 7.0.

To analyze the expression of *MCAP* gene in *P. pastoris*, a set of recombinant plasmids carrying either the partial or the whole *MCAP* gene, was cloned into the *P. pastoris* expression vector pGAPZα-A. The secreted MCAP forms were separated by SDS-PAGE. The analysis showed that the recombinant yeast X-33/pGAPZα+MCAP-5 expressed two forms of MCAP when cultivated in YPD medium at pH 5.0 (Figure 3, lane 2, Figures 4A and 5) as well as the recombinant yeast X-33/pGAPZα+SyMCAP-6 (Figures 4B, and 5, lanes, 6 and 7). The molecular mass of the largest protein was 37 kDa while that of the smallest protein was 33 kDa. Both proteins seem to have 2.5 kDa of the additional amino acids of the C-terminal polyhistidine tag since the molecular mass was distinctly higher than 30 kDa of the single MCAP from *M. circinelloides* (Figure 3, lane 7). It was confirmed that, MCAP was expressed in two forms; one glycosylated and the other non-glycosylated. Incubation of the MCAP with endo H resulted in the decrease in the apparent molecular weight (Figure 4A), giving values identical to those of the authentic MCAP from *M. circinelloides*.

None of the other recombinants analyzed in this study was able to produce MCAP. It is possible that *P. pastoris* containing plasmid pGAPZα+MCAP (data not shown) was unable to cleave the *MCAP* gene intron sequence. Such a situation has been shown in *S. cerevisiae* that did not secrete *R. niveus* aspartic proteinase as it contained an intron sequence [19]. In the case of strain containing pGAPZα+MCAP-2 and pGAPZα+MCAP-3 (Figure 3, lanes 4, 5, respectively), the start codon of α-MF secretion signal and start codon of *MCAP* are each very close to the promoter, which might have caused some inhibition of transcription. The unsuccessful result of X-33/pGAPZα+MCAP-SP (Figure 3, lanes 6) could have been due to the deleted part of MCAP proenzyme sequence, which is very important for its conversion to the mature form.

### Effect of glucose concentration, temperature and initial pH on MCAP production

#### Glucose concentration

**Figure 6:**
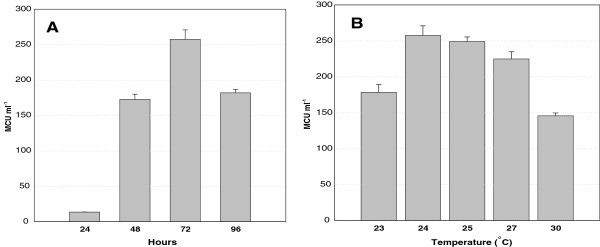
**Extracellular production of MCAP from recombinant *P. pastoris *X- 33/pGAPZα+MCAP-5. A)** Time course in YPD medium containing 4% glucose at 24°C. **B)** Production of aspartic proteinase after 72 hours in YPD medium containing 4% glucose. The values shown are the mean activity with standard deviation obtained from three sets of experiments.

The activity of the MCAP produced by the recombinant X-33/pGAPZα+MCAP-5 grown in two concentrations of glucose as the sole carbon source in the YPD medium at pH 5.0 and 24°C was compared. When glucose was used at 20 g L^-1^ the relative activity of MCAP decreased to 40% compared to a glucose concentration of 40 g L^-1^*.* The time course of MCAP production by X-33/pGAPZα+MCAP-5 (Figures 5 and 6A) showed that after 24, 48, 72 and 96 h of growth the activity of the crude enzyme was 13 (7 mg L^-1^), 172 (54 mg L^-1^), 257 (110 mg L^-1^) and 181 MCU mL^-1^ (100 mg L^-1^), respectively. Therefore, it was concluded that the maximum enzyme activity of 257 MCU mL-^1^ of fermentation broth was after approximately 72 h of cultivation when culture cells were in their late exponential growth phase and decreased after 96 h when the cells reached the stationary phase. The increase in activity was due to the quality of enzyme produced (Figures 5 and 6A). Furthermore, when the original *MCAP* gene was adapted to the optimal codon usage of *P. pastoris*, the expression of aspartic proteinase in *P. pastoris (X-*33/pGAPZα+SyMCAP-6) increased by nearly 40%. The amount of MCAP produced after 72 h of cultivation was 186 mg L^-1^ and the maximum enzyme activity was 580 MCU. The amount of MCAP in the culture supernatant was estimated as the difference between the calculated proteins produced from the recombinant *P. pastoris* and wild-type *P. pastoris*, as well as by considering the band intensities on SDS-PAGE.

### Effect of temperature

The recombinant *P. pastoris* with the original *MCAP* gene was grown for 72 h at 23, 24, 25, 27 and 30°C and the enzyme activity of 178, 260, 248, 224 and 145 MCU mL^-1^, was obtained, respectively. Temperature seemed to affect MCAP expression in *P. pastoris* and the optimum temperature for the MCAP production by X-33/pGAPZα+MCAP-5 was found to be 24°C (Figure 6B).

### Effect of pH

The effect of pH on the activity of the recombinant enzyme produced in the culture medium incubated at 24°C for 4 days and supplemented with 40 g L^-1^ glucose was investigated. When the initial pH of the culture medium was 7 instead of 5, the relative enzyme activity was reduced to 55.6% while the levels of protein expressed decreased only by 5%. Additionally, regardless of the temperature, X-33/pGAPZα+MCAP-5 and X-33/pGAPZα+SyMCAP-6 produced four forms of the recombinant protein with molecular weights of 44, 40, 37 and 33 kDa when the initial pH value of the medium was 7 (Figure 5). After the cultivation period the pH of the cultivation media decreased from 7 to 6.3 thus confirming previous observations made for *Mucor* sp. Rennin. The model for the processing of prepro-MPR, a zymogen of *Mucor* sp. Rennin expressed in *S. cerevisiae*, where it was demonstrated that prepro-MPR matured under the acidic pH [20]. This suggests that the MCAP forms of 44 and 40 kDa were also glycosylated and inactive. However, they were converted to the mature proteins with a molecular weight of 37 and 33 kDa at pH 5.0.

### Characterization of MCAP

#### Optimum pH

The MCAP proteins were tested for milk clotting activity at various pH values. The maximum activity in all proteins was observed at pH 3.6. At pH 7.0 the activity decreased drastically and the damage was irreversible. For this result, the histidine-tagged recombinant protein (MCAP) was not purified by affinity chromatography on immobilized metal (IMAC).

### Optimum temperature and thermal stability

The MCAP activity was determined as a function of temperature from 35 to 65°C. It was found that the activity was highest at 60°C regardless of protein type. In some cases, activity began to decrease at temperatures above 50°C. For this reason, thermostability was tested by incubating the enzyme samples at temperatures ranging from 55 to 60°C. The non-purified MCAPs retained 75% of their activity at 55°C and 40–60% of its activity was retained at 60°C after 60 min incubation at pH 3.6 (Table 3). Also, it was found that the purified MCAP could not retain much activity compared to the non-purified protein. Purified MCAPs retained less than 40% of their enzyme activity at 55°C after 30 min incubation at pH 3.6 while the commercial preparation of *R. miehei* showed 85% of residual activity under the same conditions. Therefore, the purified MCAPs have a remarkable difference in thermal stability in comparison to the commercial protease from *R. miehei*. The enzymes that are sensitive to heat treatment are considered suitable for milk clotting and for that reason; MCAP is a potential alternative for industrial utilization.

**Table 3:** Characteristics of the purified recombinant aspartic proteinase (MCAP)

Molecular mass* (kDa)	**Optimum temperature (°C)**	**Optimum pH**	Thermostability ** (%)
33 & 37	60	3.6	40

*Enzyme having (2.5 kDa) the additional amino acids of the C-terminal polyhistidine tag.

**Thermal stability of the enzymes at 55°C, for 30 min.

### Proteolytic activity of purified MCAP

The ratio of milk clotting activity to proteolytic activity (MCA/PA) of MCAP was compared to the value observed for commercial rennet preparation. The higher the MCA/PA ratio the more desirable the enzyme is during cheese making. Table 4 shows the MCAP ratio of about 20, which is below the calculated ratio for chymosin preparation.

**Table 4:** **Clotting and proteolytic activities of *P. pastoris *** **and *R. miehei *** **aspartic protease**

**Sample**	**Milk clotting activity MCA (U/μg)**	**Proteolytic activity PA (U/μg)**	**Ratio MCA/PA**
MCAP	137	7.02 ± 0.28	19.5 ± 0.79
*R. miehei*	311	11.11 ± 0.27	28.0 ± 0.68

Results shown are the means of three sets of experiments.

## Conclusion

The expression of MCAP under the control of the constitutive *GAP* promoter was investigated. *P. pastoris* was shown to be a good host for the production of MCAP protein and the novel MCAP was efficiently secreted into the medium to concentrations exceeding 180 mg L^-1^. Similar results were obtained by Yamashita and coworkers who cloned the *M. pusillus* Rennin gene in *S. cerevisiae* cells [21]. *P. pastoris* secreted two forms of MCAPs where one form was glycosylated while the other was non-glycosylated and similar to the authentic aspartic proteinase of the *M. circinelloides.* The observation was correlated to the presence of an N-glycosylation site Asn-Phe-Thr at position Asn331 of the amino acid sequence of MCAP. Previous reports show that aspartic proteinases expressed in *S. cerevisiae*[21,22] and *Aspergillus nidulans* were secreted as single protein bands and in most cases glycosylated [23]. However, previous observations have shown that a mutant strain defective in N–glycosylation process of *M. pusillus* excreted three glycoforms of *M. pusillus* proteins [24]. *P. pastoris* strain SMD1168 transformed with pGAPZα+MCAP-5 also excreted two forms of MCAPs (unpublished data). Interestingly, the MCAP protein contains the sequon located towards the C-terminus (Asn331 according to the MCAP of 394 amino acid residues). Therefore, *P. pastoris* can possibly excrete two forms of MCAPs. Results obtained by Shakin-Eshleman *et al.*, suggest that a particular amino acid at the X position of an Asn-X-Ser sequon is critical for Core-Glycosylation Efficiency (CGE) [25]. They found that the substitution of the amino acid X with Phe, increases the efficiency of core glycosylation. In fact, MCAP contains Phe at the X position of the sequon. The result showed that the density of the band representing glycosylated recombinant protein was more intense than the recombinant non-glycosylated protein.

To elucidate the origin of the two types of proteins, mutations could be generated at the Asn-X-Ser sequon of MCAP.

### Nucleotide sequence accession numbers

The sequences for *MCAP* determined in this article have been submitted to GenBank under accession numbers JQ906105 and JQ906106.

## Competing interests

Authors declare that they have no competing of interests.

## Authors’ contributions

JAGS, MK and MFL have designed the work. JAGS carried out the experiment. JAGS, MK and MFL analyzed the data and contributed for the statistical analysis. JAGS, MK and MFL wrote the manuscript and reviewed the manuscript critically. All the authors have read the article and approved the final manuscript.
